# Crystal structure of 2-(4-methyl­phen­yl)-4*H*-1,3-benzo­thia­zine

**DOI:** 10.1107/S205698901402725X

**Published:** 2015-01-03

**Authors:** N. C. Sandhya, Chandra, G. P. Suresha, N. K. Lokanath, M. Mahendra

**Affiliations:** a Department of Studies in Chemistry, Manasagangotri, University of Mysore, Mysore 570 006, India; b Department of Studies in Physics, Manasagangotri, University of Mysore, Mysore 570 006, India

**Keywords:** crystal structure, benzo­thia­zine derivative, biological properties, C—H⋯π inter­actions

## Abstract

In the title compound, C_15_H_13_NS, the thia­zine ring adopts a boat conformation. The dihedral angle between the planes of the benzene ring of the benzo­thia­zine unit and the tolyl ring is 19.52 (9)°. In the crystal, mol­ecules are linked by weak C—H⋯π inter­actions into a tape structure along the *b-*axis direction.

## Related literature   

For the biological importance of benzo­thia­zine derivatives, see: Ahmad *et al.* (2010 ▸); Gupta *et al.* (2002 ▸); Lazzeri *et al.* (2001 ▸); Parveen *et al.* (2014 ▸); Zia-ur-Rehman *et al.* (2006 ▸).
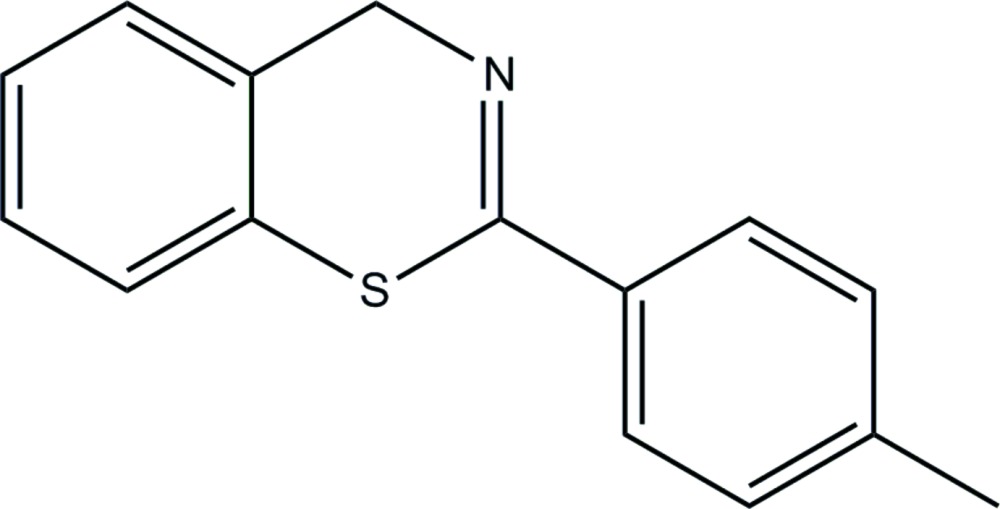


## Experimental   

### Crystal data  

C_15_H_13_NS
*M* *_r_* = 239.33
Monoclinic, 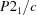

*a* = 15.1241 (9) Å
*b* = 6.0111 (4) Å
*c* = 14.3212 (9) Å
β = 110.268 (2)°
*V* = 1221.36 (13) Å^3^
*Z* = 4
Cu *K*α radiation
μ = 2.13 mm^−1^
*T* = 293 K
0.30 × 0.25 × 0.20 mm

### Data collection  

Bruker X8 Proteum diffractometer
10379 measured reflections
1988 independent reflections
1890 reflections with *I* > 2σ(*I*)
*R*_int_ = 0.041

### Refinement  

*R*[*F* ^2^ > 2σ(*F* ^2^)] = 0.036
*wR*(*F* ^2^) = 0.102
*S* = 1.05
1988 reflections
156 parameters
H-atom parameters constrained
Δρ_max_ = 0.18 e Å^−3^
Δρ_min_ = −0.20 e Å^−3^

### 

Data collection: *APEX2* (Bruker, 2009 ▸); cell refinement: *SAINT* (Bruker, 2009 ▸); data reduction: *SAINT*; program(s) used to solve structure: *SHELXS97* (Sheldrick, 2008 ▸); program(s) used to refine structure: *SHELXL97* (Sheldrick, 2008 ▸); molecular graphics: *PLATON* (Spek, 2009 ▸); software used to prepare material for publication: *SHELXL97*.

## Supplementary Material

Crystal structure: contains datablock(s) global, I. DOI: 10.1107/S205698901402725X/is5385sup1.cif

Structure factors: contains datablock(s) I. DOI: 10.1107/S205698901402725X/is5385Isup2.hkl

Supporting information file. DOI: 10.1107/S205698901402725X/is5385Isup3.cml

**Fig. 1.**
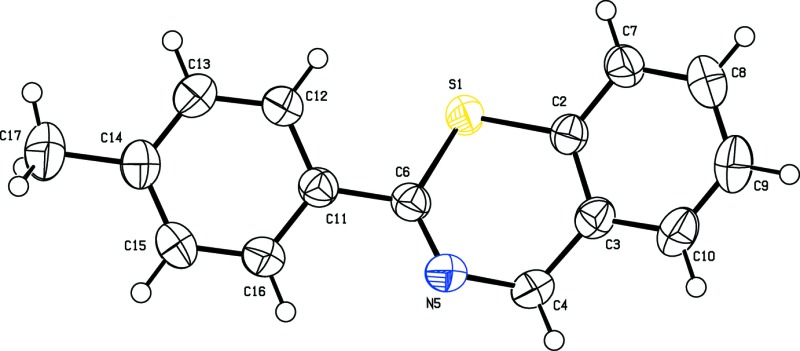
The molecular structure of the title compound with 50% probability displacement ellipsoids. H atoms are drawn as spheres of arbitrary radii.

**Fig. 2.**
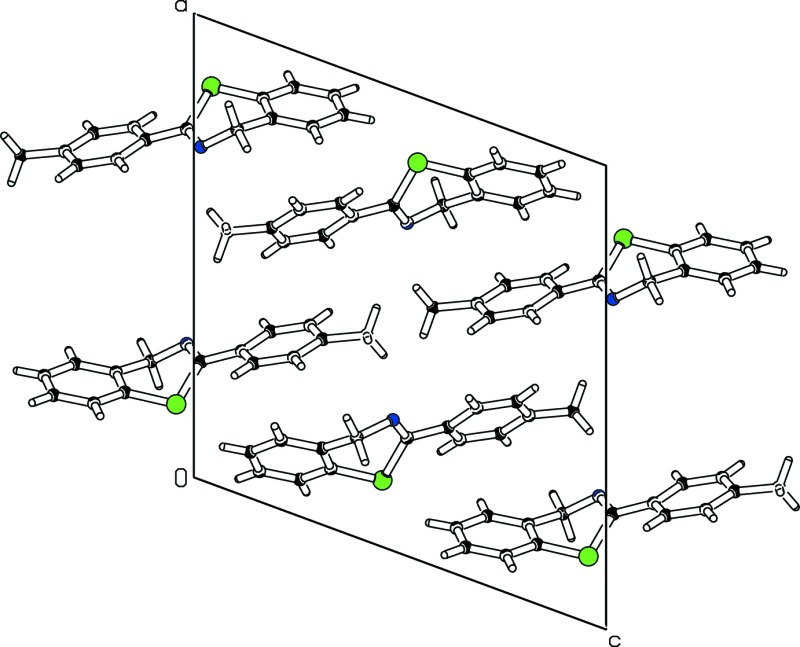
A packing diagram of the title compound viewed along the b axis.

. DOI: 10.1107/S205698901402725X/is5385fig1.tif
The mol­ecular structure of the title compound with 50% probability displacement ellipsoids. H atoms are drawn as spheres of arbitrary radii.

b . DOI: 10.1107/S205698901402725X/is5385fig2.tif
A packing diagram of the title compound viewed along the *b* axis.

CCDC reference: 1039090

Additional supporting information:  crystallographic information; 3D view; checkCIF report

## Figures and Tables

**Table 1:** Hydrogen-bond geometry (, ) *Cg* is the centroid of the C3/C2/C7C10 ring.

*D*H*A*	*D*H	H*A*	*D* *A*	*D*H*A*
C7H7*Cg* ^i^	0.93	2.75	3.485(2)	136

Symmetry code: (i) 
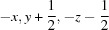
.

## supplementary crystallographic information

### S1. Comment

Benzothiazines have been found to posses versatile biological activities, such as analgesic (Gupta *et al.*, 2002), anti bacterial (Zia-ur-Rehman *et al.*, 2006) and antioxidant activities (Ahmad *et al.*, 2010). Also, benzothiazine derivatives have shown activities for the treatment of asthmatic therapy (Lazzeri *et al.*, 2001). Recently, 1,2-benzothiazine-1,1-dioxide and its derivatives were reported as aldose reductase inhibitors (Parveen *et al.*, 2014). With this potential background of benzothiazine derivatives, we have synthesized the title compound to study its crystal structure.

In the title compound (Fig. 1), the mean plane of the benzothiazine moiety (S1/C2/C7–C10/C3/C4/N5/C6) makes a dihedral angle of 19.52 (9)° with the benzene ring (C11–C16). The central thiazine ring adopts a boat conformation with puckering parameter Q = 0.5848 (16) Å and φ = 183.41 (17)°, and the maximum deviation found on the puckered atom at C6 is -0.170 (2) Å. There are no classic hydrogen bonds. Instead, a weak C—H···π interaction is observed (C7—H7···*Cg*^i^; *Cg*: C3/C2/C7–C10; Table 1). The molecular packing exhibits layered stacking when viewed down the *b* axis as shown in Fig. 2.

### S2. Experimental

4-Methyl-*N*-[(phenylthio)methyl]benzamide was heated with POCl_3_ (10 ml) on an oil bath for 1 h. The reaction mixture was cooled by treated with ice, neutralized with Na_2_CO_3_, and extracted with dichloromethane. The combined extracts were dried (Na_2_SO_4_) and solvent was evaporated off. The residue was recrystalized from hot ethanol to get crystals of the title compound.

### S3. Refinement

All H atoms were positioned geometrically and allowed to ride on their parent atom, with C—H = 0.93–0.97 Å, and with *U*_iso_(H) = 1.2–1.5*U*_eq_(C).

### Crystal data

**Table:**

C_15_H_13_NS	*F*(000) = 504
*M**_r_* = 239.33	*D*_x_ = 1.302 Mg m^−^^3^
Monoclinic, *P*2_1_/*c*	Cu *K*α radiation, λ = 1.54178 Å
Hall symbol: -P 2ybc	Cell parameters from 1988 reflections
*a* = 15.1241 (9) Å	θ = 3.1–64.6°
*b* = 6.0111 (4) Å	µ = 2.13 mm^−^^1^
*c* = 14.3212 (9) Å	*T* = 293 K
β = 110.268 (2)°	Block, light yellow
*V* = 1221.36 (13) Å^3^	0.30 × 0.25 × 0.20 mm
*Z* = 4	

### Data collection

**Table:**

Bruker X8 Proteum diffractometer	1890 reflections with *I* > 2σ(*I*)
Radiation source: Bruker MicroStar microfocus rotating anode	*R*_int_ = 0.041
Helios multilayer optics monochromator	θ_max_ = 64.6°, θ_min_ = 3.1°
Detector resolution: 10.7 pixels mm^-1^	*h* = −17→16
φ and ω scans	*k* = −3→6
10379 measured reflections	*l* = −16→16
1988 independent reflections	

### Refinement

**Table:**

Refinement on *F*^2^	Secondary atom site location: difference Fourier map
Least-squares matrix: full	Hydrogen site location: inferred from neighbouring sites
*R*[*F*^2^ > 2σ(*F*^2^)] = 0.036	H-atom parameters constrained
*wR*(*F*^2^) = 0.102	*w* = 1/[σ^2^(*F*_o_^2^) + (0.0608*P*)^2^ + 0.3174*P*] where *P* = (*F*_o_^2^ + 2*F*_c_^2^)/3
*S* = 1.05	(Δ/σ)_max_ = 0.001
1988 reflections	Δρ_max_ = 0.18 e Å^−^^3^
156 parameters	Δρ_min_ = −0.20 e Å^−^^3^
0 restraints	Extinction correction: *SHELXL97* (Sheldrick, 2008), FC^*^=KFC[1+0.001XFC^2^Λ^3^/SIN(2Θ)]^-1/4^
Primary atom site location: structure-invariant direct methods	Extinction coefficient: 0.0063 (7)

### Special details

**Table:**

Geometry. Bond distances, angles *etc*. have been calculated using the rounded fractional coordinates. All su's are estimated from the variances of the (full) variance-covariance matrix. The cell e.s.d.'s are taken into account in the estimation of distances, angles and torsion angles
Refinement. Refinement on *F*^2^ for ALL reflections except those flagged by the user for potential systematic errors. Weighted *R*-factors *wR* and all goodnesses of fit *S* are based on *F*^2^, conventional *R*-factors *R* are based on *F*, with *F* set to zero for negative *F*^2^. The observed criterion of *F*^2^ > σ(*F*^2^) is used only for calculating -*R*-factor-obs *etc*. and is not relevant to the choice of reflections for refinement. *R*-factors based on *F*^2^ are statistically about twice as large as those based on *F*, and *R*-factors based on ALL data will be even larger.

### Fractional atomic coordinates and isotropic or equivalent isotropic displacement parameters (Å^2^)

**Table:**

	*x*	*y*	*z*	*U*_iso_*/*U*_eq_	
S1	0.85647 (3)	0.07017 (7)	0.54278 (3)	0.0440 (2)	
N5	0.71804 (10)	−0.2361 (2)	0.51727 (10)	0.0482 (5)	
C2	0.87171 (11)	0.0058 (3)	0.66754 (11)	0.0383 (5)	
C3	0.83561 (11)	−0.1929 (3)	0.68916 (12)	0.0422 (5)	
C4	0.78453 (13)	−0.3459 (3)	0.60484 (13)	0.0519 (6)	
C6	0.74506 (11)	−0.0644 (2)	0.48288 (12)	0.0394 (5)	
C7	0.92024 (12)	0.1532 (3)	0.74273 (12)	0.0461 (5)	
C8	0.93470 (13)	0.0983 (3)	0.84074 (13)	0.0554 (6)	
C9	0.90031 (14)	−0.0989 (4)	0.86320 (14)	0.0595 (7)	
C10	0.85010 (12)	−0.2426 (3)	0.78801 (13)	0.0534 (6)	
C11	0.68996 (11)	0.0295 (3)	0.38404 (12)	0.0399 (5)	
C12	0.70649 (13)	0.2415 (3)	0.35473 (13)	0.0494 (6)	
C13	0.66238 (14)	0.3119 (3)	0.25780 (14)	0.0548 (6)	
C14	0.59980 (13)	0.1777 (3)	0.18745 (13)	0.0520 (6)	
C15	0.58033 (14)	−0.0299 (3)	0.21806 (14)	0.0577 (6)	
C16	0.62461 (13)	−0.1037 (3)	0.31386 (13)	0.0501 (6)	
C17	0.55616 (18)	0.2545 (4)	0.08089 (15)	0.0766 (8)	
H4A	0.83080	−0.42520	0.58460	0.0620*	
H4B	0.75040	−0.45520	0.62890	0.0620*	
H7	0.94270	0.28720	0.72730	0.0550*	
H8	0.96770	0.19490	0.89160	0.0660*	
H9	0.91090	−0.13580	0.92930	0.0710*	
H10	0.82580	−0.37380	0.80390	0.0640*	
H12	0.74760	0.33660	0.40080	0.0590*	
H13	0.67520	0.45350	0.23960	0.0660*	
H15	0.53630	−0.12110	0.17280	0.0690*	
H16	0.61070	−0.24440	0.33200	0.0600*	
H17A	0.56570	0.41170	0.07730	0.1150*	
H17B	0.48980	0.22310	0.05730	0.1150*	
H17C	0.58520	0.17760	0.04030	0.1150*	

### Atomic displacement parameters (Å^2^)

**Table:**

	*U*^11^	*U*^22^	*U*^33^	*U*^12^	*U*^13^	*U*^23^
S1	0.0437 (3)	0.0489 (3)	0.0373 (3)	−0.0101 (2)	0.0113 (2)	0.0032 (2)
N5	0.0492 (8)	0.0407 (8)	0.0485 (8)	−0.0079 (6)	0.0092 (6)	0.0025 (6)
C2	0.0358 (8)	0.0401 (8)	0.0380 (8)	0.0031 (7)	0.0117 (6)	0.0024 (7)
C3	0.0395 (8)	0.0430 (9)	0.0442 (9)	0.0027 (7)	0.0145 (7)	0.0064 (7)
C4	0.0598 (11)	0.0388 (10)	0.0518 (10)	−0.0056 (8)	0.0127 (8)	0.0080 (8)
C6	0.0402 (9)	0.0373 (9)	0.0396 (8)	−0.0010 (6)	0.0126 (7)	−0.0030 (6)
C7	0.0434 (9)	0.0466 (10)	0.0428 (9)	0.0001 (7)	0.0081 (7)	−0.0009 (7)
C8	0.0515 (11)	0.0685 (12)	0.0395 (9)	0.0058 (9)	0.0074 (8)	−0.0055 (8)
C9	0.0585 (11)	0.0820 (14)	0.0385 (9)	0.0096 (10)	0.0174 (9)	0.0103 (9)
C10	0.0514 (10)	0.0612 (12)	0.0497 (10)	0.0034 (9)	0.0203 (8)	0.0177 (9)
C11	0.0392 (8)	0.0393 (9)	0.0405 (9)	0.0006 (7)	0.0128 (7)	−0.0015 (7)
C12	0.0546 (10)	0.0414 (10)	0.0449 (9)	−0.0046 (8)	0.0078 (8)	−0.0011 (7)
C13	0.0610 (11)	0.0452 (10)	0.0533 (10)	0.0016 (8)	0.0135 (9)	0.0084 (8)
C14	0.0480 (10)	0.0587 (11)	0.0440 (9)	0.0060 (8)	0.0091 (8)	0.0053 (8)
C15	0.0520 (11)	0.0627 (12)	0.0461 (10)	−0.0094 (9)	0.0014 (8)	−0.0031 (9)
C16	0.0487 (10)	0.0453 (10)	0.0500 (10)	−0.0075 (8)	0.0092 (8)	0.0003 (8)
C17	0.0780 (15)	0.0877 (16)	0.0494 (11)	0.0016 (12)	0.0035 (10)	0.0156 (11)

### Geometric parameters (Å, º)

**Table:**

S1—C2	1.7635 (16)	C14—C15	1.388 (3)
S1—C6	1.7967 (17)	C14—C17	1.510 (3)
N5—C4	1.465 (2)	C15—C16	1.375 (3)
N5—C6	1.2705 (19)	C4—H4A	0.9700
C2—C3	1.392 (3)	C4—H4B	0.9700
C2—C7	1.391 (2)	C7—H7	0.9300
C3—C4	1.502 (2)	C8—H8	0.9300
C3—C10	1.388 (2)	C9—H9	0.9300
C6—C11	1.483 (2)	C10—H10	0.9300
C7—C8	1.384 (2)	C12—H12	0.9300
C8—C9	1.377 (3)	C13—H13	0.9300
C9—C10	1.384 (3)	C15—H15	0.9300
C11—C12	1.391 (3)	C16—H16	0.9300
C11—C16	1.393 (3)	C17—H17A	0.9600
C12—C13	1.382 (3)	C17—H17B	0.9600
C13—C14	1.379 (3)	C17—H17C	0.9600
			
C2—S1—C6	99.02 (8)	N5—C4—H4B	109.00
C4—N5—C6	118.70 (15)	C3—C4—H4A	109.00
S1—C2—C3	119.34 (12)	C3—C4—H4B	109.00
S1—C2—C7	119.55 (13)	H4A—C4—H4B	108.00
C3—C2—C7	121.11 (15)	C2—C7—H7	120.00
C2—C3—C4	118.61 (14)	C8—C7—H7	120.00
C2—C3—C10	118.43 (15)	C7—C8—H8	120.00
C4—C3—C10	122.95 (16)	C9—C8—H8	120.00
N5—C4—C3	114.95 (14)	C8—C9—H9	120.00
S1—C6—N5	123.71 (13)	C10—C9—H9	120.00
S1—C6—C11	114.10 (11)	C3—C10—H10	120.00
N5—C6—C11	121.96 (15)	C9—C10—H10	120.00
C2—C7—C8	119.27 (17)	C11—C12—H12	120.00
C7—C8—C9	120.18 (17)	C13—C12—H12	120.00
C8—C9—C10	120.36 (17)	C12—C13—H13	119.00
C3—C10—C9	120.62 (17)	C14—C13—H13	119.00
C6—C11—C12	122.43 (15)	C14—C15—H15	119.00
C6—C11—C16	119.58 (15)	C16—C15—H15	119.00
C12—C11—C16	117.75 (16)	C11—C16—H16	120.00
C11—C12—C13	120.59 (17)	C15—C16—H16	120.00
C12—C13—C14	121.68 (17)	C14—C17—H17A	110.00
C13—C14—C15	117.54 (17)	C14—C17—H17B	109.00
C13—C14—C17	120.58 (18)	C14—C17—H17C	109.00
C15—C14—C17	121.87 (18)	H17A—C17—H17B	109.00
C14—C15—C16	121.47 (18)	H17A—C17—H17C	109.00
C11—C16—C15	120.87 (17)	H17B—C17—H17C	110.00
N5—C4—H4A	109.00		
			
C6—S1—C2—C3	31.75 (16)	S1—C6—C11—C12	−20.0 (2)
C6—S1—C2—C7	−148.81 (15)	S1—C6—C11—C16	154.33 (14)
C2—S1—C6—N5	−30.02 (16)	N5—C6—C11—C12	165.34 (17)
C2—S1—C6—C11	155.37 (12)	N5—C6—C11—C16	−20.4 (3)
C6—N5—C4—C3	47.9 (2)	C2—C7—C8—C9	−0.8 (3)
C4—N5—C6—S1	−6.7 (2)	C7—C8—C9—C10	−0.8 (3)
C4—N5—C6—C11	167.49 (15)	C8—C9—C10—C3	1.6 (3)
S1—C2—C3—C4	−0.1 (2)	C6—C11—C12—C13	171.49 (18)
S1—C2—C3—C10	178.66 (14)	C16—C11—C12—C13	−2.9 (3)
C7—C2—C3—C4	−179.54 (17)	C6—C11—C16—C15	−172.56 (18)
C3—C2—C7—C8	1.6 (3)	C12—C11—C16—C15	2.0 (3)
C7—C2—C3—C10	−0.8 (3)	C11—C12—C13—C14	1.0 (3)
S1—C2—C7—C8	−177.85 (15)	C12—C13—C14—C15	1.8 (3)
C2—C3—C4—N5	−43.9 (2)	C12—C13—C14—C17	−177.1 (2)
C10—C3—C4—N5	137.37 (18)	C13—C14—C15—C16	−2.7 (3)
C2—C3—C10—C9	−0.8 (3)	C17—C14—C15—C16	176.1 (2)
C4—C3—C10—C9	177.91 (19)	C14—C15—C16—C11	0.8 (3)

### Hydrogen-bond geometry (Å, º)

*Cg* is the centroid of the C3/C2/C7–C10 ring.

**Table:**

*D*—H···*A*	*D*—H	H···*A*	*D*···*A*	*D*—H···*A*
C7—H7···*Cg*^i^	0.93	2.75	3.485 (2)	136

Symmetry code: (i) −*x*, *y*+1/2, −*z*−1/2.
