# Supplementary figures and images for: Crystal structure of 2-(4-methyl­phen­yl)-4H-1,3-benzo­thia­zine
Source: Acta Crystallogr E Crystallogr Commun. 2015 Jan 3;71(Pt 2):o74. doi: 10.1107/S205698901402725X (PMC4384615; doi:10.1107/S205698901402725X)

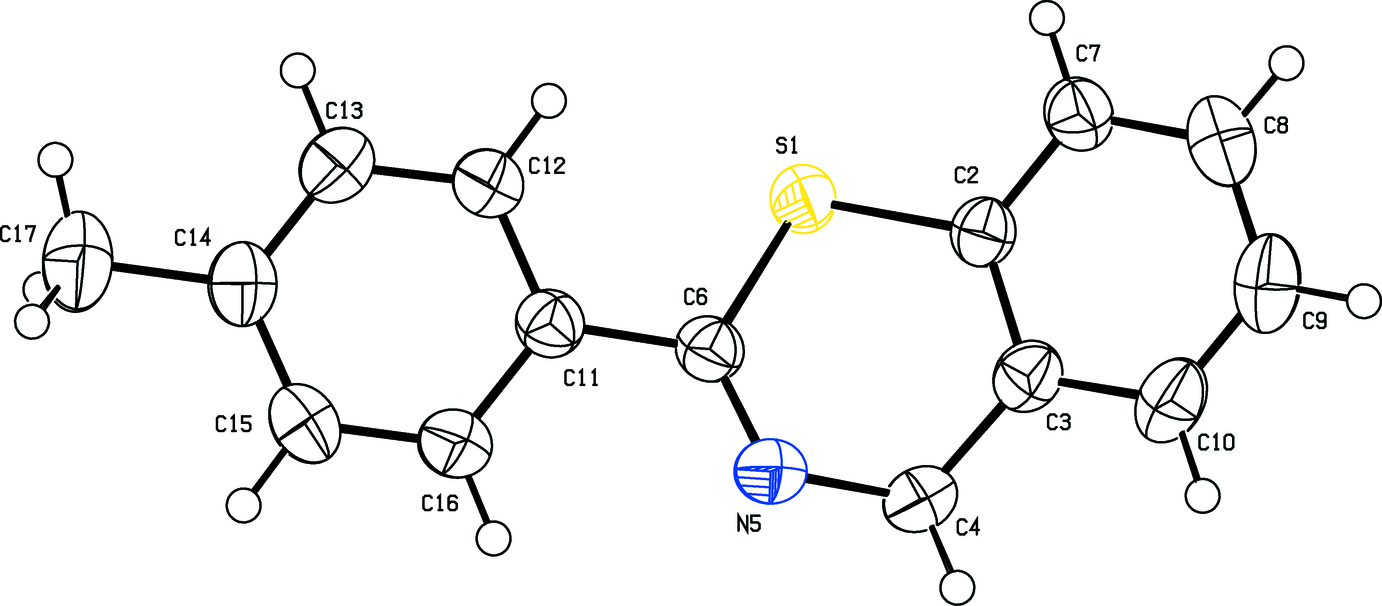

Supplement: Supplementary file 4 [file e-71-00o74-fig1.tif]

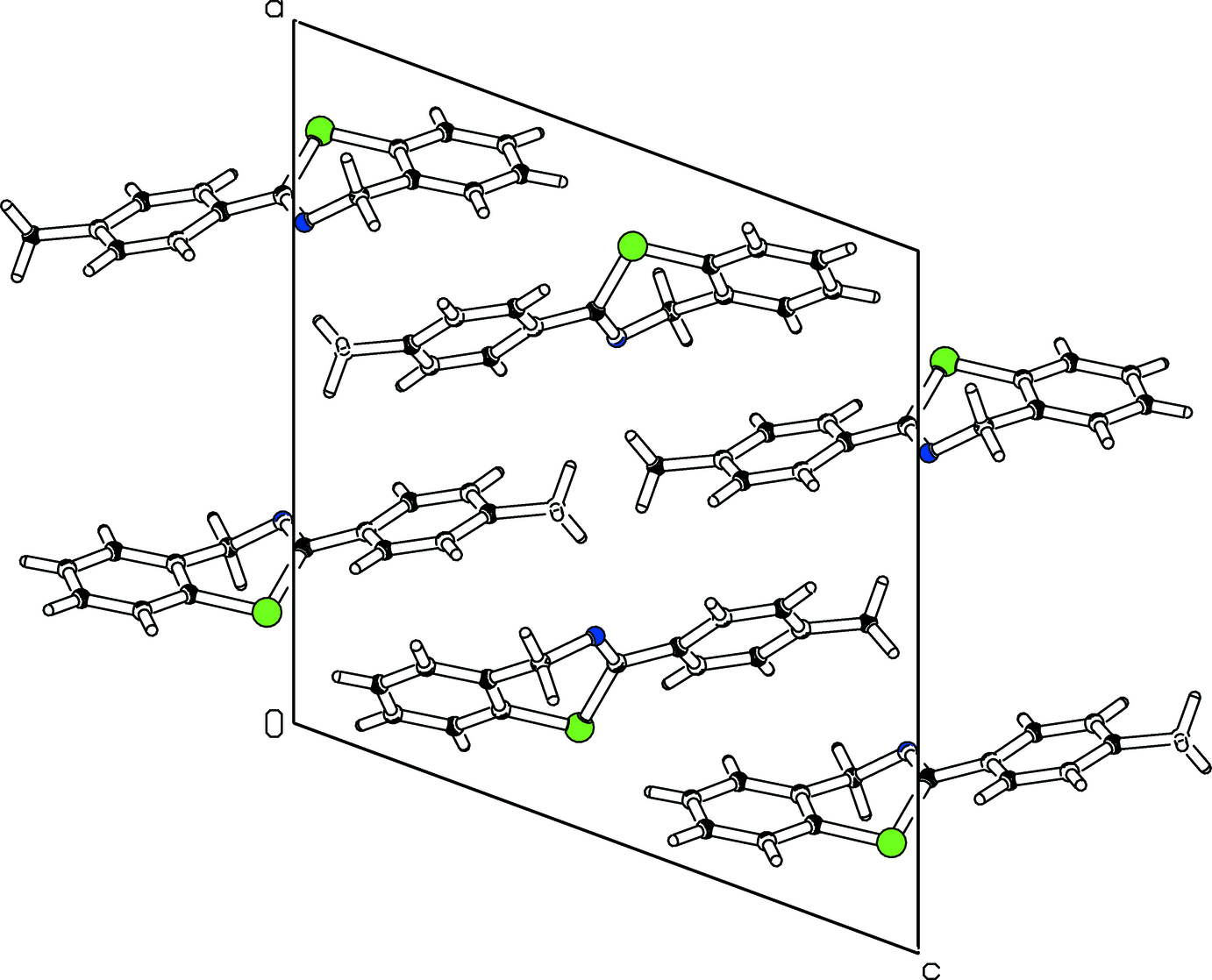

Supplement: Supplementary file 5 [file e-71-00o74-fig2.tif]
